# A Novel High-Throughput 3D Screening System for EMT Inhibitors: A Pilot Screening Discovered the EMT Inhibitory Activity of CDK2 Inhibitor SU9516

**DOI:** 10.1371/journal.pone.0162394

**Published:** 2016-09-13

**Authors:** Kazuya Arai, Takanori Eguchi, M. Mamunur Rahman, Ruriko Sakamoto, Norio Masuda, Tetsuya Nakatsura, Stuart K. Calderwood, Ken-ichi Kozaki, Manabu Itoh

**Affiliations:** 1 SCIVAX Life Sciences, Inc., Kawasaki, Kanagawa, Japan; 2 SCIVAX USA, Inc., Woburn, Massachusetts, United States of America; 3 Department of Radiation Oncology, Beth Israel Deaconess Medical Center, Harvard Medical School, Boston, Massachusetts, United States of America; 4 Department of Dental Pharmacology, Okayama University Graduate School of Medicine, Dentistry and Pharmaceutical Sciences, Okayama, Japan; 5 Advanced Research Center for Oral and Craniofacial Sciences, Okayama University Dental School / Graduate School of Medicine, Dentistry and Pharmaceutical Sciences, Okayama, 700–8525, Japan; 6 Division of Cancer Immunotherapy, Exploratory Oncology Research & Clinical Trial Center, National Cancer Center, Kashiwa, Chiba, Japan; University of Colorado Boulder, UNITED STATES

## Abstract

Epithelial-mesenchymal transition (EMT) is a crucial pathological event in cancer, particularly in tumor cell budding and metastasis. Therefore, control of EMT can represent a novel therapeutic strategy in cancer. Here, we introduce an innovative three-dimensional (3D) high-throughput screening (HTS) system that leads to an identification of EMT inhibitors. For the establishment of the novel 3D-HTS system, we chose NanoCulture Plates (NCP) that provided a gel-free micro-patterned scaffold for cells and were independent of other spheroid formation systems using soft-agar. In the NCP-based 3D cell culture system, A549 lung cancer cells migrated, gathered, and then formed multiple spheroids within 7 days. Live cell imaging experiments showed that an established EMT-inducer TGF-β promoted peripheral cells around the core of spheroids to acquire mesenchymal spindle shapes, loss of intercellular adhesion, and migration from the spheroids. Along with such morphological change, EMT-related gene expression signatures were altered, particularly alteration of mRNA levels of *ECAD/CDH1*, *NCAD/CDH2*, *VIM* and *ZEB1/TCF8*. These EMT-related phenotypic changes were blocked by SB431542, a TGF-βreceptor I (TGFβR1) inhibitor. Inside of the spheroids were highly hypoxic; in contrast, spheroid-derived peripheral migrating cells were normoxic, revealed by visualization and quantification using Hypoxia Probe. Thus, TGF-β-triggered EMT caused spheroid hypoplasia and loss of hypoxia. Spheroid EMT inhibitory (SEMTIN) activity of SB431542 was calculated from fluorescence intensities of the Hypoxia Probe, and then was utilized in a drug screening of EMT-inhibitory small molecule compounds. In a pilot screening, 9 of 1,330 compounds were above the thresholds of the SEMTIN activity and cell viability. Finally, two compounds SB-525334 and SU9516 showed SEMTIN activities in a dose dependent manner. SB-525334 was a known TGFβR1 inhibitor. SU9516 was a cyclin-dependent kinase 2 (CDK2) inhibitor, which we showed also had an EMT-inhibitory activity. The half maximal inhibitory concentration (IC_50_) of SB-525334 and SU9516 were 0.31 μM and 1.21 μM, respectively, while IC_50_ of SB431542 was 2.38 μM. Taken together, it was shown that this 3D NCP-based HTS system was useful for screening of EMT-regulatory drugs.

## Introduction

Cell-based assays for a development of anti-cancer drugs have been mostly performed in monolayer culture condition [1]. On the one hand, widely attached cells have been easily handled on such two-dimensional (2D) cell culture plates/dishes, and thus, the 2D cell culture methods have been suitable for analysis of particular molecules in detail. On the other hand, the 2D cell culture have been known to enhance integrin signaling, thus to mask some of the biological activities of tumor cells [2]. Therefore, it has been implicated that physical contact of cells with tissue culture plates might result in artificial alteration of tumor cells. In contrast, other researchers have reported that three-dimensional (3D) cell cultures can replicate the intratumor microenvironment [3–5]. These findings suggest the importance of 3D cell culture conditions for evaluation of the cellular phenotype and of chemical compounds and/or cytokines. Recently, Kumar et. al. reported that mesenchymal transition of non-small cell lung carcinoma (NSCLC) cell lines were much more efficiently induced on 3D cell culture condition than 2D [6]. These reported persuaded us to develop a novel 3D screening system.

NanoCulture Plate (NCP) is a cell/tissue culture plate with patterned nano-scale grids on the plate base [7]. This mogul field on the NCPs restricts cells from sprawling on the base and enables tumor cells to migrate more than the monolayer layer cell culture system [8]. Cells migrate from a scaffold to another scaffold on the grid in the NCPs more actively than cells cultured on 2D plate. The increased migration and lesser attachment of tumor cells on the NCPs concurrently enable tumor cells to form 3D spheroids in FBS-containing medium. Another advantage of the NCPs in tumor and cell biology is that NCPs is useful without gel materials [7]. Cells cultured on NCPs have high proliferation activity as same as on 2D cell culture conditions. The NCP-based cell-culture system can also be useful for selection of malignant cells that can grow in anchoring-independent condition as shown in soft-agar. The NCP-based 3D cell-culture method is suitable for assessing morphological change, which is a key phenomenon in tumor progression. Epithelial-mesenchymal transition (EMT) is a morphological change of tissues/cells from an epithelial form to a fibroblast-like mesenchymal form. This biological event has been shown to participate in physiological development, tissue regeneration and tumor progression, particularly in tumor budding and metastasis [9–11]. In addition to the morphological changes, key biomarkers in the steps of EMT have been established including cell adhesion molecules and, transcription factors and kinases [12]. Tumor cells along with progression of EMT assume a spindled shape, lose desmosomes and adherence junctions, and express mesenchymal molecules such as microtubule vimentin and a zinc-finger E-box transcription factor ZEB-1/TCF8 [9,13]. The expression of EMT biomarkers has been reported to be correlated with drug resistance [14–18]. Vice versa, inhibition of EMT may interfere with tumor progression and drug resistance. Transforming Growth Factor-β (TGF-β) signaling have been shown to be involved in a large number of cellular processes including EMT [19]. TGF-β signaling inhibitors have been evaluated in oncologic pre-clinical and clinical studies [20,21].

In this paper, we firstly developed an *in vitro* EMT model on NCP-based 3D cell culture system and then established a novel 3D high-throughput screening (HTS) system for screening of EMT inhibitors. In order to quantitate sizes and intercellular adhesion of 3D spheroid, we also utilized Hypoxia Probes, an agent that is composed of a phosphorescent light–emitting iridium complexes, whose emitting energy is quenched by oxygen molecules in normoxic condition [22]. Using such materials and system, we screened novel EMT-inhibitory compounds from a library of 1,330 pharmacologically active compounds and then obtained screening hits that indeed inhibited EMT.

## Materials and Methods

### Reagents and antibodies

Human recombinant TGF-β2 (R&D systems, Minneapolis, MN) stock solution (20 μg/mL) was prepared with 0.1% BSA/4 mM HCL according to manufacturer’s procedure, and used at a final concentration of 5 ng/mL An ALK inhibitor, SB431542 was obtained from Stemgent (Cambridge, MA). SB-525334 was from Sigma-Aldrich (St. Louis, MO). SU9516 was from Merck Millipore (Billerica, MA). These compound were dissolved in DMSO at 10 mM as a stock solution and diluted when used. Antibodies used were: E-cadherin (mouse monoclonal, clone 36/E-Cadherin), obtained from BD Biosciences (San Jose, CA). An anti-β-actin antibody (mouse monoclonal, clone AC-15) was obtained from Sigma–Aldrich (St. Louis, MO). All secondary horseradish peroxidase-conjugated antibodies were obtained from Jackson ImmunoResearch (West Grove, PA). Alexa 488-conjugated secondary antibody was obtained from Abcam (Cambridge, MA).

### Cell culture, control of EMT status

We used A549 human lung carcinoma cell line and 4 human pancreatic cancer cell lines: Capan-2 well-differentiated adenocarcinoma derived from a primary tumor, AsPC-1, PANC-1, and MIA PaCa-2 poorly differentiated adenocarcinoma derived from primary tumor. These cell lines were obtained from American Type Culture Collection and maintained on plastic tissue culture plates in Dulbecco's modified Eagle's medium (DMEM, Nisshin EM, Tokyo, Japan) supplemented with 10% inactivated fetal bovine serum. Induction of EMT was performed in DMEM containing 5% fetal bovine serum with 5 ng/mL TGF-β2 (R&D Systems, MN). SB431542 was used as positive control at 10 μM. Negative control is the vehicle, DMSO. NanoCulture Plate (SCIVAX Life Sciences, Kanagawa, Japan) was used as 3D cell culture system. Cells were seeded in 96-well plate at a density of 10,000 cells/well or in 384 well plate at a density of 3,000 cells/well, respectively.

### Live-Cell Imaging

After 3 days culturing, spheroids were treated with or without TGF-β2. Then time-lapse images of these spheroids were filmed using a BioStation CT (Nikon Co., Tokyo, Japan) every 2 hours for 4 days.

### RNA extraction and quantitative RT-PCR

Total RNA was isolated with the RNeasy Plus Mini Kit, according to the manufacturer’s instructions (Qiagen, Hilden, Germany). Reverse transcription was performed with PrimeScript™ RT reagent Kit (TaKaRa, Shiga, Japan) including a mixture of oligo dT and random primers. Real-time PCR was carried out with the synthesized cDNA, SYBR^®^ Premix Ex Taq™ II (Tli RNaseH Plus) and primer sets in a Thermal Cycler Dice Real Time System (TaKaRa, Shiga, Japan). Expression data were normalized to the geometric mean of housekeeping gene *TBP* to control the variability in expression levels and were analyzed using the 2 -ΔΔCT method described by Livak and Schmittgen [23].

The following primer pairs were used: *TBP* forward, 5’-TGCTGCGGTAATCATGAGGATA, *TBP* reverse, 5’-TGAAGTCCAAGAACTTAGCTGGAA (TaKaRa Shiga, Japan), *CHD1* forward, 5'-ATTGCAAATTCCTGCCATTC, *CDH1* reverse TCCTCCGAAGAAACAGCAAG, *CDH2* forward 5'-AGGTTTGCCAGTGTGACTCC, *CDH2* reverse 5'-CCACAAACATCAGCACAAGG, *VIM* forward 5'-AGACAGGTGCAGTCCCTCAC, *VIM* reverse 5'-GCTTCAACGGCAAAGTTCTC, *ZEB1/TCF8* forward 5'-GGAAAGCGCTTCTCACACTC, and *ZEB1/TCF8* reverse 5'-GTCACGTTCTTCCGCTTCTC.

### Immunoblotting

Cells were lysed within Cell Lysis Buffer (10×) (Cell Signaling Technologies) according to manufacturer’s protocol. And 5 μg protein samples were separated by SDS–PAGE 10% and transferred to a 0.45 μm pore size PVDF membrane (EMD Millipore, MA). Blocking and antibody reaction steps were carried out in skim milk solution. After antibody incubation, peroxidase activity was detected via chemiluminescence (ECL reagent; Bio-Rad, Hercules, CA).

### Hypoxia imaging and quantification

Hypoxia Probe (SCIVAX USA, Woburn, MA) were dissolved in DMSO at 1 mM as a stock solution and stored at -20°C in the dark. The stock solution was diluted with culture medium and added to the culturing medium at a final concentration of 2 μM per well. One day after the addition, spheroid images and the fluorescent intensities of Hypoxia Probe were obtained with a cell imaging equipment, Celigo (Nexelom, Lawrence, MA). Integrated fluorescent intensities (IFI) from the probes in a whole well were calculated by multiplying the number of segmented spheroids by an average of mean intensity because multiple spheroids were included in one well.

### Pilot screening targeted to 1,330 pharmacologically active compounds

We chose 10 μM SB431542 as a positive control. Dimethyl sulfoxide (DMSO), a vehicle of compounds, was used as a negative control. Each compound was diluted with DMSO at 400 μM in Polypropylene 384 well V-bottom plates as daughter plates. Positive/negative controls were added in the each daughter plates. Pre-cultured cells were harvested by 0.05% Trypsin/0.5 mM EDTA solution and seeded into NCP 384 well plate at a concentration of 3,000 cells/well/45 μL medium. Three days later, drugs of daughter plates with or without TGF-β2 were diluted with culture medium. Then the solution was added into the wells in 5 μL each (final concentration of chemical compounds, DMSO and TGF-β2 were 1 μM, 0.25% and 5 ng/mL, respectively). After the treatment for 3 days, spheroids/cells were treated with 2 μM Hypoxia Probe for overnight. The signal intensity was measured with Celigo. Then finally, cell viability was evaluated by luminescence ATP assay, as described herein below.

### Calculation of Spheroid EMT inhibitory activity

Spheroid EMT inhibitory (SEMTIN) activity of each target compounds were calculated from IFIs of target compounds and positive/negative-controls for adequate evaluation of these target compounds. The formula is as follow:

$$SEMTIN\;activity=\frac{{IFI}_{\left(w/o\;TGF-\beta 2\right)}–{IFI}_{\left(with\;TGF-\beta 2\right)}\times \alpha }{\lambda }\times 100\;(\%)$$

α is a reciprocal EMT inducible efficiency on negative control, explained as *IFI*_*(with TGF-β2)*_ *÷ IFI*_*(w/o TGF-β2)*_.

*λ* is an amount of inhibited EMT on positive control, explained as *IFI*_*(w/o TGF-β2)*_ − *IFI*_*(with TGF-β2)*_ *×* α

### Cell Survival Assay

The viability of the cells was estimated by quantification of the ATP present using a CellTiter-Glo (CTG) Luminescent Cell Viability Assay (Promega Co., Madison, WI), a reagent with a luminescent readout that reflects cell viability via the measurement of ATP metabolism. A half of culture supplement (25 μL) were removed and an equal volume of CTG solution was added to each well, and then suspended by liquid handler. The plate was rocked on a shaker for 10 min and incubated for an additional 20 min at 37°C. Luminescent measurements were done on Infinite M200 (Tecan, Männedorf, Switzerland), plate reader. For assays measuring toxicity effects, all values were normalized to the mock-treated conditions.

### Immunofluorescence staining for E-cadherin and quantification

Cells were seeded onto NCP or 8 well chamber slide (BD Biosciences, San Diego, CA). After treatment with TGF-β2 and with/without drugs, spheroids were directly fixed on NCPs or 8-well chamber slide by adding 1/10 volume of 40% formaldehyde solution into the culture medium for 15 min. Cells were permeabilized with 0.2% Tween-20 in PBS(-) for 10 min at RT. Then, spheroids were incubated with the primary antibody for E-cadherin diluted in PBS for overnight at 4°C, followed by incubation with the secondary antibody for 1 hour at RT. And the nuclei were counterstained with DAPI. Immunoreactivities were visualized with imaging cytometer Celigo for 3D, or confocal-microscopy FLUOVIEW FV10i (Olympus, Tokyo, Japan) for 2D. Fluorescence signal of E-cadherin and DAPI in a whole well were integrated. To calculate E-cadherin expression level per cell, the integrated E-cadherin fluorescent signal was divided by the integrated DAPI signal, and then compared with the vehicle control (0.1% BSA/4 mM HCL and DMSO)

## Results

### Development of *in vitro* 3D EMT model on NanoCulture Plate

**Fig 1:**
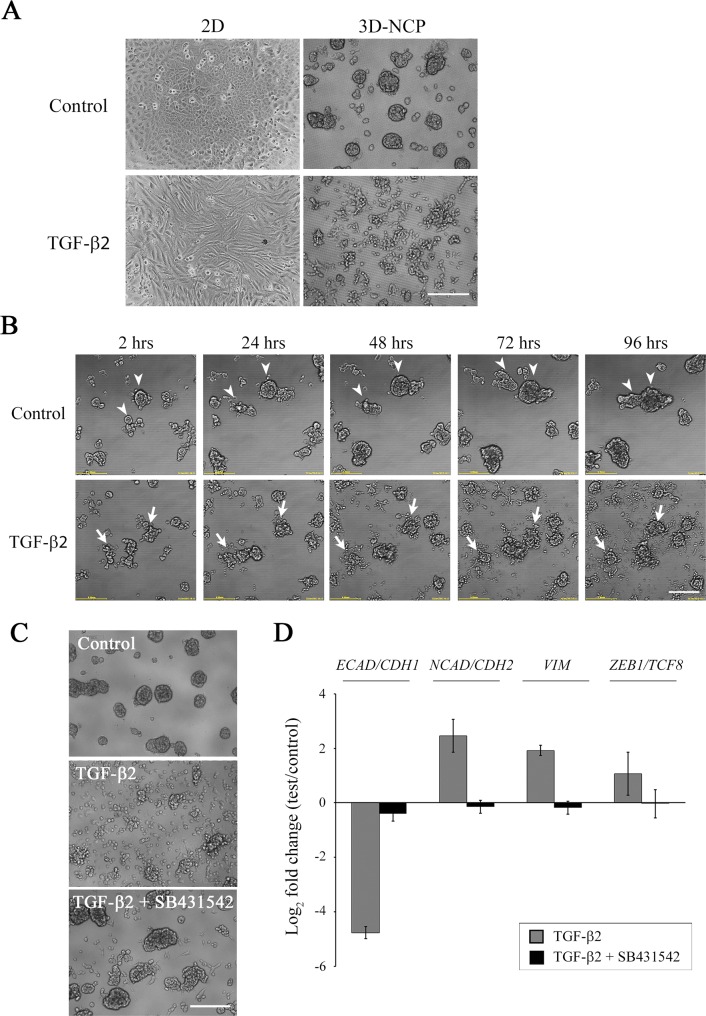
Epithelial to mesenchymal transition (EMT) on NanoCulture Plate (NCP). (A) A549 cells were grown in 2D or 3D-NCP conditions with or without TGF-β2 for 4 days. The cells treated with TGF-β2 on 2D condition appeared to present fibroblastic spindle shape. In the 3D-NCP condition, TGF-β2-treated spheroid cells appeared to spread out from spheroids, and spheroid sizes were reduced. (B) Representative images of kinetic morphology change. Cells were pre-cultivated on NCP for 3 days and then treated with TGF-β2 for 4 days. Arrowheads indicate spheroids that fused into one spheroid. Arrow indicate spheroid collapse induced by the addition of TGF-β2. Scale bars, 200 μm. Live-cell imaging video is available in S1 and S2 Movies. (C) A549 cells were cultured with or without TGF-β2 only or TGF-β2 + SB431542 for 4 days. Representative images were shown. Scale bar, 200 μm. (D) mRNA levels of *ECAD/CDH1*, *NCAD/CDH2*, *VIM* and *ZEB1/TCF8* were quantified with real-time qRT-PCR analyses. Values were normalized to *TBP* levels, and then were shown as the mean of fold change to vehicle control ± SD of three independent experiments

In order to develop the 3D *in vitr*o EMT model, we used NanoCulture Plate on which tumor cells could easily migrate and form spheroids. On the NCPs, a human NSCLC cell line, A549, rapidly migrated, aggregated and formed spheroids of varying size (Fig 1A, lower left). TGF-β has been known to induce EMT [24,25]. We treated A549 cells with TGF-β2 on 2D and 3D plate and evaluated whether TGF-β2 induces EMT in 3D-NCP culture condition. The cells treated with TGF-β2 on 2D condition appeared to adopt fibroblastic spindle shapes (Fig 1A, upper right). In contrast, cells treated with TGF-β2 did not form spheroids in the 3D-NCP condition. These cells formed loose clusters with scarce intercellular adhesion. A few single cells had spindle shapes (Fig 1A, lower right). For a greater understanding of the spheroid morphological change induced by the TGF-β2 treatment, a time-lapse observation was then conducted. We observed that TGF-β2 treatment prompted cells to spread out from spheroids, declined intercellular adherence and reduced spheroid sizes, in contrast to non-treatment spheroids (Fig 1B and S1 and S2 Movies). Thus, the A549 cells formed 3D spheroids on the NCPs, but the cells fail to form spheroids after TGF-β2 treatment. Treatment with TGF-β2 appeared to inhibit interspheroid fusions and to increase cellular budding and subsequent release from the spheroids, which made the spheroid surface rough. Such changes around the surface of the spheroids appeared to block the formation of larger spheroids and finally to reduce sizes of the aggregated spheroids compared to the control.

Next, we confirmed the effect of TGF-β receptor type I (TGFβR1) inhibitor SB431542 on this change of spheroid morphology. SB431542 inhibited the spheroid morphological change caused by TGF-β2 (Fig 1C). Furthermore, we examined epithelial and mesenchymal gene expressions in the A549 cells treated with TGF-β2 only and TGF-β2 plus SB431542 compared to non-treated control on the NCPs. For qRT-PCR, *TBP* was chosen as an internal control gene (S1 Fig). The mRNA level of *ECAD/CDH1*, the epithelial marker, was decreased by treatment with TGF-β2. In contrast, mRNA levels of *NCAD/CDH2*, *VIM* and *ZEB1/TCF8* were increased by the treatment of TGF-β2 (Fig 1D). These alterations in EMT-related gene expression were inhibited by the addition of SB431542 (Fig 1C and 1D). These results showed that the spheroid morphological change, which was induced with TGF-β2, was caused as the result of EMT at a molecular level. And as a result, we have effectively established an *in vitro* EMT model on NCP.

### Correlation between hypoxia and EMT model on NCP

**Fig 2:**
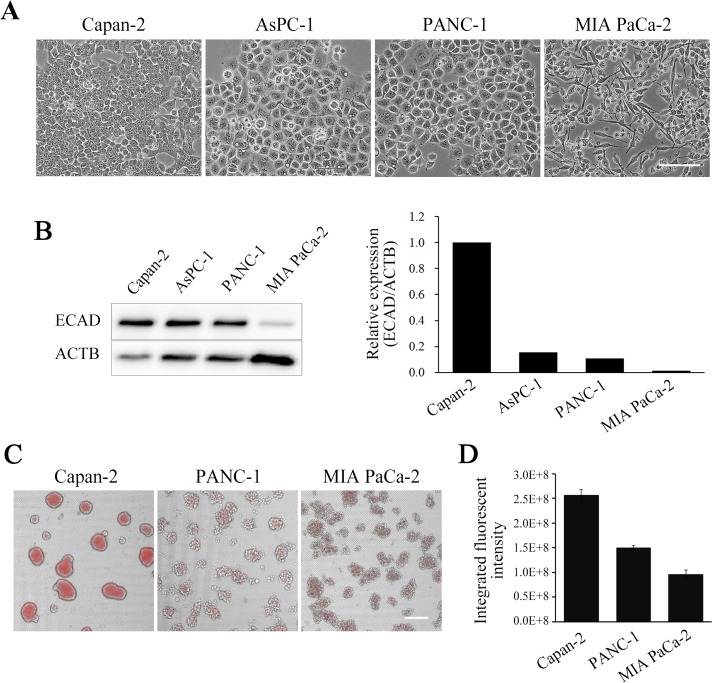
Hypoxia level of spheroids was positively correlated with E-cadherin level in pancreatic cancer cell lines. (A) Representative phase contrast images of four types of pancreatic cancer cell lines cultured on 2D condition. Scale bar, 100 μm. (B) Western blotting analysis of E-cadherin in the 4 types of pancreatic cancer cell lines cultured on NCPs for 7 days. β-actin was examined as the loading control. Signal intensities were quantified, and relative values were shown on the graph. (C) Representative hypoxic images of pancreatic cancer cell lines cultured on NCPs. Hypoxia Probe was added to the culture medium and visualized under fluorescence microscopy, shown in red. The fluorescence and bright field images were merged. Scale bar, 200 μm. (D) Hypoxia levels among the cell lines. Integrated fluorescent intensities (IFI) of the Hypoxia Probe taken in spheroids were calculated as described in Materials and Methods. N = 3. Data are mean ± SD.

It has been shown that tumor progression is a process where well-differentiated normal epithelial or ductal cells de-differentiates into mesenchymal-like cells. Many de-differentiation changes in tumor cells have been explained by the loss of the epithelial markers, such as E-cadherin, and by the gain of mesenchymal markers, such as N-cadherin [26,27]. E-cadherin, the inter-cellular adhesion molecule is essential not only for maintenance of epithelial status but also for spheroid formation and morphology (cell-cell connection) [28–30]. Hypoxic status in spheroids have been reported as below. Sutherland et al. reported that the center of spheroids can have a more hypoxic status compared with the peripheral layers of the spheroid [31]. Wartenberg et al. reported the correlation between spheroid size and hypoxia condition [32]. Park et al. described that round and compact spheroids exhibited highly hypoxic inner core [33]. In relation to these evidences, we examined E-cadherin expression levels, spheroid morphology, and hypoxic status of several pancreatic cancer cell lines with different differentiation levels on the 3D-NCP system [25,26]. To investigate correlativity between differentiation levels and spheroid features, we first observed cellular shapes of Capan-2, AsPC-1, PANC-1 and MIA PaCa-2 pancreatic cancer cell lines on 2D culture condition. Among these 4 cell lines, Capan-2 showed the most epithelial shapes, AsPC-1 and PANC-1 showed an intermediate epithelial morphology, and MIA PaCa-2 showed the most mesenchymal spindle shape in 2D culture condition (Fig 2A). Next, we compared E-cadherin expression levels of these cell lines. The expression level of E-cadherin was the highest in Capan-2 and the lowest in MIA PaCa-2 (Capan-2>AsPC-1, PANC-1>MIA PaCa-2) (Fig 2B), indicated that Capan-2 was epithelial, MIA PaCa-2 was mesenchymal, and AsPC-1 and PANC-1 were intermediates. We next cultured three cell lines on NCPs and examined the spheroid morphology and hypoxic levels of these cell lines, using Hypoxia Probe After cultured for 7 days on NCPs, these cell lines formed spheroids/cell-aggregates (Fig 2C and S2 Fig). Capan-2 formed large and smooth surface spheroids, PANC-1 and MIA PaCa-2 formed small size spheroids in loose cell-to-cell adherence (Fig 2C). Thus, these cell lines formed different types of spheroids correlated with E-cadherin expression levels. Next, we evaluated integrated fluorescence intensities (IFI) of the Hypoxia Probe taken up into spheroids. Among these cell lines, Capan-2 spheroids showed the highest degree of hypoxia, PANC-1 spheroids were of intermediate degree, and MIA PaCa-2 spheroids were the most normoxic (Fig 2D). These results indicated that Capan-2 had the highest cell density per spheroid among these 3 cell lines. Taken together, these experiments indicated that the hypoxia levels of spheroids tend to be positively correlated with E-cadherin expression levels and with the morphology of spheroids with higher intercellular adhesion levels and spheroid volume.

**Fig 3:**
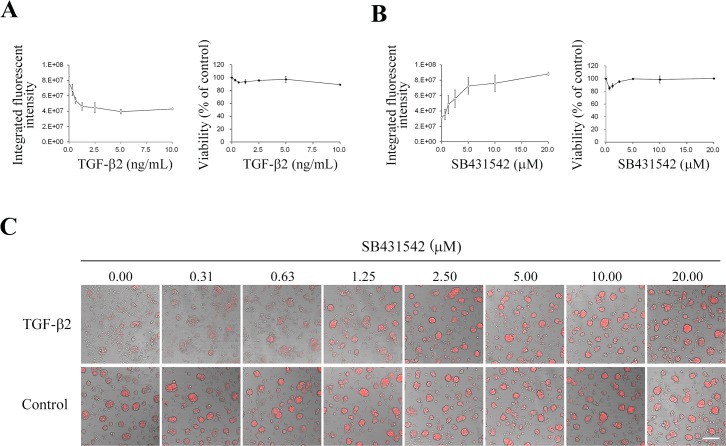
Hypoxia level of A549 spheroid was declined with TGF-β2 and elevated with SB431542. (A) A549 spheroids cultured on NCP for 3 days were stimulated with TGF-β2 for 4 days in indicated concentrations. Hypoxia Probe was then added. Integrated fluorescent intensities (IFI) of Hypoxia Probe were reduced in a dose depending manner without affecting viability. (B) A549 spheroids were treated with TGF-β2 (5 ng/mL) and indicated concentration of SB431542 for 4 days. IFI of Hypoxia Probe were increased in a dose depending manner without affecting viability. N = 3. Data are mean ± SD. (C) Representative images of hypoxic spheroids restituted by SB431542. A549 spheroids were treated with 5 ng/mL TGF-β2 and SB431542 at indicated concentrations. Hypoxia levels were visualized with Hypoxia Probe in red color Scale bars, 200 μm.

Next, in order to clarify the relation between the degrees of hypoxia and EMT, we quantitated hypoxia levels of the A549 spheroids treated with various concentrations of TGF-β2. Hypoxia levels within the cells/spheroids were decreased in a dose-dependent manner of TGF-β2 treatment (Fig 3A). Inversely, the hypoxia levels reduced with TGF-β2 were increased by the treatment with SB431542 in a dose-dependent manner (Fig 3B and 3C). Besides, the cellular viabilities were not significantly altered by the increasing concentrations of either TGF-β2 or SB431542 (Fig 3). Thus, the quantification of the hypoxia level using Hypoxia Probe may be useful as an indicator of spheroid formation along with the increase in intercellular adhesion. This assessment using the Hypoxic Probe could be helpful for assessment of EMT inhibitors by HTS.

### Screening of potential EMT inhibitors, and validation of hits

**Fig 4:**
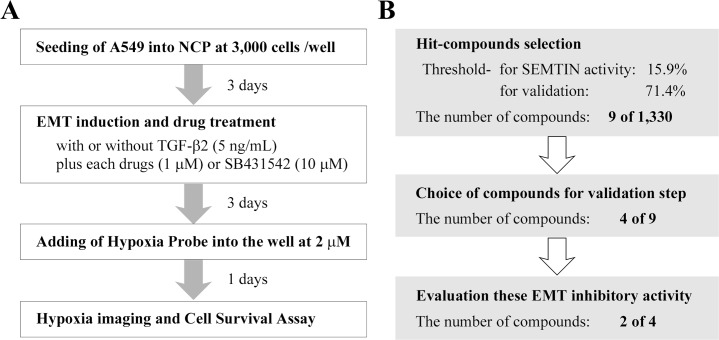
Procedures for screening and validation of EMT inhibitors. (A) A Scheme of EMT inhibitor screening using NCP and Hypoxia Probe. TGF-β2 (5 ng/mL) and drugs (1 μM) were added at day 3 after seeding of A549 cells. SB431542 was used as a positive control. Hypoxia Probe was added at day 6, and then cell viability were evaluated at day 7. (B) A schematic overview of EMT inhibitor screening of 1,330 compounds. We provided thresholds as more than 15.9% Cell viability and more than 71.4% SEMTIN activity. Nine compounds were over the thresholds. Of these, 4 compounds were selected for validation. Of these, 2 compounds showed the SEMTIN activity.

**Fig 5:**
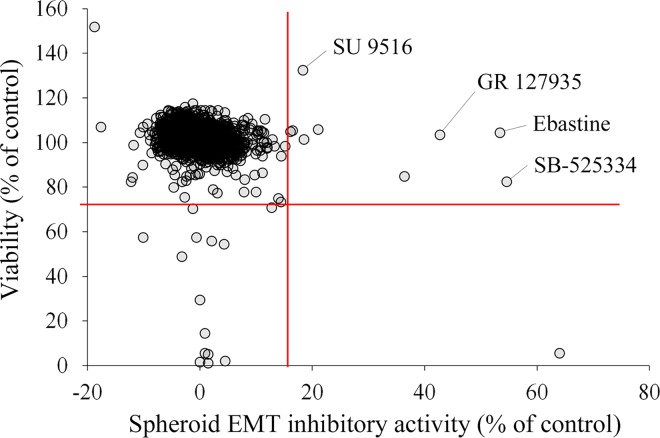
Pilot screening of EMT inhibitors from 1,330 pharmacologically active compounds. Scatter plots was shown. The x-axis indicates the EMT inhibitory efficiency, which was calculated as described in Materials and Methods. These data were the mean value of the independent two experiments. SB-525334 (a potent ALK5/type I TGF-β-receptor kinase inhibitor), SU9516 (a cyclin-dependent kinase 2 (CDK2) inhibitor), GR 127935 (A selective and potent 5-HT1B/1D serotonin receptor antagonist) and Ebastine (a histamine H1-receptor antagonist) as indicated were selected for validation.

Finally, we developed a unique assay system for screening EMT inhibitors using the *in vitro* EMT model and monitoring hypoxia condition of inside spheroid (Fig 4A). For a proof-of-concept of this assay system, we conducted a pilot screening using a chemical library of 1,330 pharmacologically active compounds consist of LOPAC 1280 and Prestwick Chemical Library, and the strategy of this screening was shown in Fig 4B. IFI can be affected by auto-fluorescence of the compounds and of cells including dead cells induced by compounds. These factors can encompass false negative and false positive, respectively. In order to evaluate the effect of compounds on the spheroid hypoplasia precisely, spheroid EMT inhibitory (SEMTIN) activity of each target compound was calculated from IFI as described in Materials and Methods. As a result of the screening, 9 compounds were selected as hit compounds (the thresholds of SEMTIN activity and viability are 15.9% (mean + 3SD) and 71.4% (mean − 3SD), respectively (Fig 4B and Fig 5). SB-525334 which is a potent ALK5/type I TGF-β-receptor kinase inhibitor [34], was included in the hit compounds. This result validated this system worked as a screening assay for EMT inhibitors.

**Fig 6:**
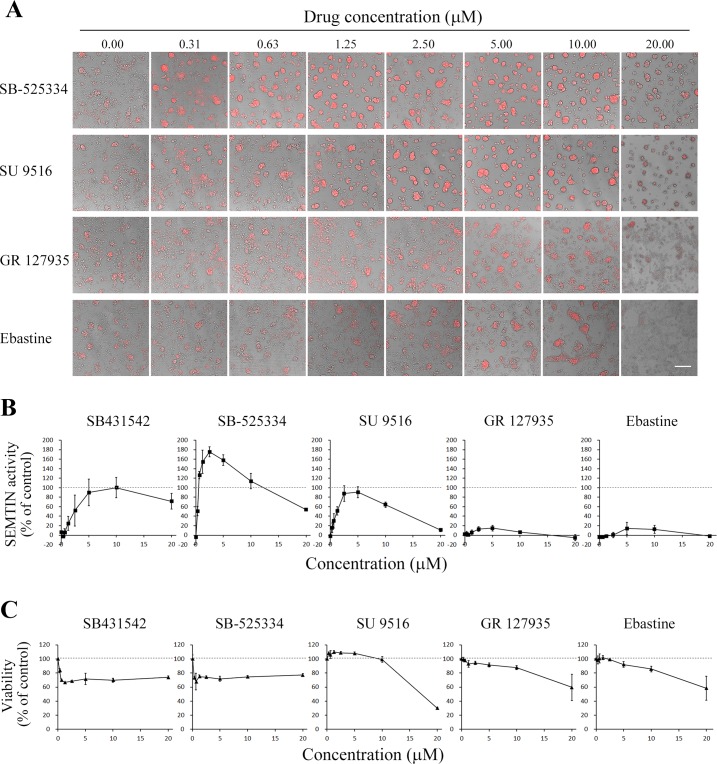
Dose dependency of SEMTIN activity of screened compounds. (A) Representative images of spheroids that was altered by drugs. Spheroids were treated with 5 ng/mL TGF-β2 and each drug at indicated concentrations. Hypoxia levels of spheroids were measured and shown in red. Scale bars, 200 μm. (B) Dose-dependent SEMTIN activities of the compounds. SB431542 is a positive control. SEMTIN activity IC_50_ of SB-525334, SU9516, and SB431542 were 0.31 μM, 1.21 μM, and 2.38 μM, respectively. N = 4. Data are mean ± SD. (C) Cellular viability under the exposure of compounds.

We then chose 4 compounds, SB-525334, SU9516, GR 127935 hydrochloride and Ebastine, for further validation. In the validation of the screening, we evaluated the dose-dependent manner of these compounds both on SEMTIN activity and cell viability (Fig 6). (Effects of these drugs to spheroids, which were not treated with TGF-β2 were showed in S3 Fig). A549 cells formed spheroids in treatment with SB-525334 and SU9516 in dose-dependent manners (Fig 6A). The SEMTIN activities of these compounds were also dose dependent (Fig 6B). These data implied that these two compounds inhibited EMT. The SEMTIN activities of each compound were calculated in respect of 10 μM positive control (SB431542). The SEMTIN activity IC_50_ of SB-525334 and SB431542 were 0.31 μM and 2.38 μM, respectively (Fig 6B). Thus, the identified SB-525334 showed much higher SEMTIN activity than the control SB431542. The SEMTIN activity IC_50_ value of SU9516 was 1.21 μM, which was comparable to the value of SB431542 (Fig 6B) while SU9516 inhibited EMT at lower concentration (5 μM or less) and reduced cell viability at higher concentration (over 10 μM) compared to the control SB431542 (Fig 6C). These results indicated that SU9516 had both efficient SEMTIN activity and higher cytotoxicity. Although we confirmed that these two compounds had higher SEMTIN activities compared to the positive control, GR 127935 and Ebastine showed only slight SEMTIN activity approximately 15% at 5 μM compared to positive control (Fig 6B). Therefore, we acquired at least two potential candidates for novel EMT inhibitors in this pilot screening.

**Fig 7:**
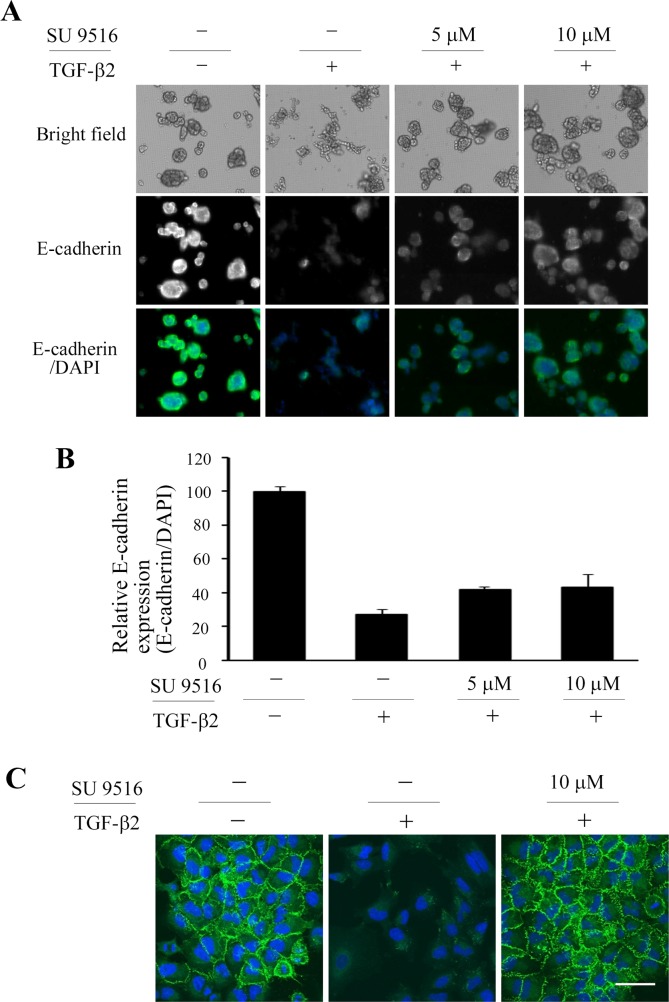
Validation of EMT inhibitory activities of screened compound, SU 9516. (A) Representative images of immunofluorescence for E-cadherin of 3D cultured cells. A549 spheroids treated with or without TGF-β2 (5 ng/mL) and SU 9516 in indicated concentration were visualized by immunofluorescence for E-cadherin (green) and with DAPI (blue). (B) E-cadherin expression levels in the 3D culture system. E-cadherin signal intensity in a whole well was integrated and divided by integrated DAPI signal. The relative values were shown as E-cadherin expression change to the vehicle control ± SD of four wells. (C) Representative images of immunofluorescence for E-cadherin of 2D cultured cells. E-cadherin at the plasma membrane (green) and DAPI counter-stained nucleus (blue) were visualized by confocal microscopy. Scale bars, 50 μm.

Furthermore, we examined whether SU 9516 could alter expression levels of EMT markers. Immunofluorescence revealed that E-cadherin expression level in A549 spheroids was reduced by treatment with TGF-β2, and SU 9516 could reinstate TGF-β2-triggered E-cadherin diminishment at a part and inhibited spheroid hypoplasia (Fig 7A and 7B). Next, we examined E-cadherin expression and localization with confocal microscopy in order to evaluate the EMT inhibitory activity of SU 9516 in 2D cell culture condition. E-cadherin expression level in A549 cells was reduced by the treatment with TGFβ2. SU 9516 appeared to inhibit the TGFβ-triggered disappearance of E-cadherin on the cell surface (Fig 7C). These results indicated that SU 9516, which was one of hit compounds from the pilot screening of EMT inhibitors, indeed had EMT inhibitory activity.

## Discussion

Several efforts at discovering anti-cancer drug including the EMT inhibiting concept have been conducted [18,20]. Chua et al. reported a development of a method screening EMT inhibitor by calculating cell motility under 2D cell culture condition [35]. It is known that 3D cell culture models are superior at recapitulating *in vivo* like growth and differentiation of tissues as compared to 2D models [36]. Therefore, some researchers have tried to develop 3D-based assay systems. Aref et al. reported an another assay system using a microfluidic device in coculture tumor cells with human umbilical vascular endothelial cells (HUVECs), in which system cell motility in 3D hydrogel can be evaluated [37]. However, these systems needed a specialized kind of equipment. Li Q et al. presented the other EMT inhibitor screening method, which does not need any special equipment. Their system was based on the evaluation of vimentin, a classic mesenchymal marker, expressed in MDA-MB-231 breast carcinoma cell line in agarose/ECM-hydrogel 3D cell culture system [38]. However, all these screening methods require reporter gene transfectants. Compared to these screening methods, our novel screening system in this paper does not require transfection or complicated devices.

Analyzing expression levels of E-cadherin, N-cadherin and vimentin is a reasonable approach. However, qRT-PCR and western blotting analyses of these factors are not efficient methods for HTS of EMT inhibitors or screening of anti-cancer drug. We needed simpler, HTS-compatible, and 3D assay system for development of EMT inhibitors. We reported in this paper the simplest assay developed so far, using commercially available plates and probe. This unique 3D-NCP allows cells to migrate due to reduced adhesion to the well surface compared to popular 2D plates. This character of the 3D-NCP increases cellular motility so that cells have more chances to attach and adhere each other and form spheroids. Therefore, we were enabled to examine spheroid morphology and hypoxia status inside. Finally, we can utilize the Hypoxia Probe, a live-cell imaging probe for hypoxia sensing, to evaluate the levels of EMT in the cells. The Hypoxia Probe does not require washout step. This character of the probe allows us to perform “add-only” assays that are suitable for efficient HTS. Another advantage of the Hypoxia Probe is low toxicity. We can, therefore, do endpoint validation assays due to this character of the Hypoxia Probe. Thus, researchers can further use the cells for cross-validation such as cell viability assay and gene expression determination after the HTS screening assay.

We screened a 1,330 member chemical library to evaluate the validity of this innovative 3D HTP screening system, and at least two compounds, SB-525334 and SU9516, showed the potential EMT inhibitory activity (Figs 6 and 7). SB-525334 is known as a TGFβR1 inhibitor, and IC_50_ value of SB-525334 and SB431542 in the kinase activity were 14.3 nM [32] and 25 nM [39], respectively. Laping et al. demonstrated that SB-525334 significantly diminished tumor incidence, multiplicity, and reduced the size of leiomyoma tumors [40]. Another hit compound SU9516 showed EMT inhibitory activity indeed. However, it seemed that the activity in 3D cell culture condition was lower than 2D (Fig 7). The different activities may result from differences in intracellular signaling conditions between 3D and 2D [2,6,41]. SU 9516 was a CDK2 inhibitor involved in TGF-β signaling pathway [42,43]. It was also reported that SU9516 showed cytotoxicity to several types of cancers [44,45]. Opyrchal et al. reported that this compound selectively targeted CD44^+^/CD24^-/Low^ cancer stem cell (CSC)-like subpopulation and restored chemoresistance in inflammatory breast cancer [46]. The relation between CSC and EMT has not been clarified yet and is under investigation. However, CSC and EMT cell phenotypes overlap [18,47]. These considerations suggested SU9516 to be an intense candidate for anti-cancer/anti-metastasis therapeutic agents.

In conclusion, we have introduced a straightforward 3D culture-based HTS-compatible EMT assay. This assay system worked for *in vitro* and rapid screening of EMT inhibitors that target the TGF-β signaling pathway. Users might set up a dynamic range suitable for their own assay system when applying this method to other cell types or cytokines. This system may promote the development of drugs for tumor metastasis through discovering novel molecular targets that control EMT.

## Supporting Information

S1 Fig Stability of internal control genes for qRT-PCR among RNA samples. Expression level of three internal control genes in four RNA samples from A549 spheroids treated with 5 ng/mL TGF-β2 or the vehicle (0.1% BSA/4 mM HCL) and 10 μM SB431542 or DMSO were evaluated by qRT-PCR. TBP was the most stable gene out of 3 among 4 RNA samples. Data are mean ± SD of triplicate well.
(TIF)

S2 Fig Spheroid area of pancreas cancer cell lines. Three pancreas cancer cell lines were cultured on NCP and observed with Celigo for image analysis. Pixels of these spheroids in a whole well were measured as spheroid size with image J software, and then the mean and median were calculated. Data are mean ± SD of triplicate well.
(TIF)

S3 Fig Effect of four candidate drugs to spheroids in indicated doses. No EMT induction, only drug treated spheroids hypoxia images (red color). A549 spheroids cultured on NCP for 3 days were treated with each drugs at indicated concentrations for 4 days. In 20 μM SU 9516 treatment, spheroid sizes were small.
(TIF)

S1 Movie A549 Spheroids Morphology on NanoCulture Plate after treated with 0.1% BSA / 4 mM HCL from day 3 to day 7 without TGF-β2 as a control. After cell seeding, time-lapse images were taken every 2 hours for 4 days with BioStation CT (objective lens is 10×).
(ZIP)

S2 Movie A549 Spheroids Morphology change on NanoCulture Plate after treated with the TGF-β2, 0.1% BSA/4 mM HCL from day 3 to day 7. Then time-lapse images were taken every 2 hours for 4 days with BioStation CT objective lens is 10×).
(ZIP)
