# Supplementary figures and images for: A Novel High-Throughput 3D Screening System for EMT Inhibitors: A Pilot Screening Discovered the EMT Inhibitory Activity of CDK2 Inhibitor SU9516
Source: PLoS One. 2016 Sep 13;11(9):e0162394. doi: 10.1371/journal.pone.0162394 (PMC5021355; doi:10.1371/journal.pone.0162394)

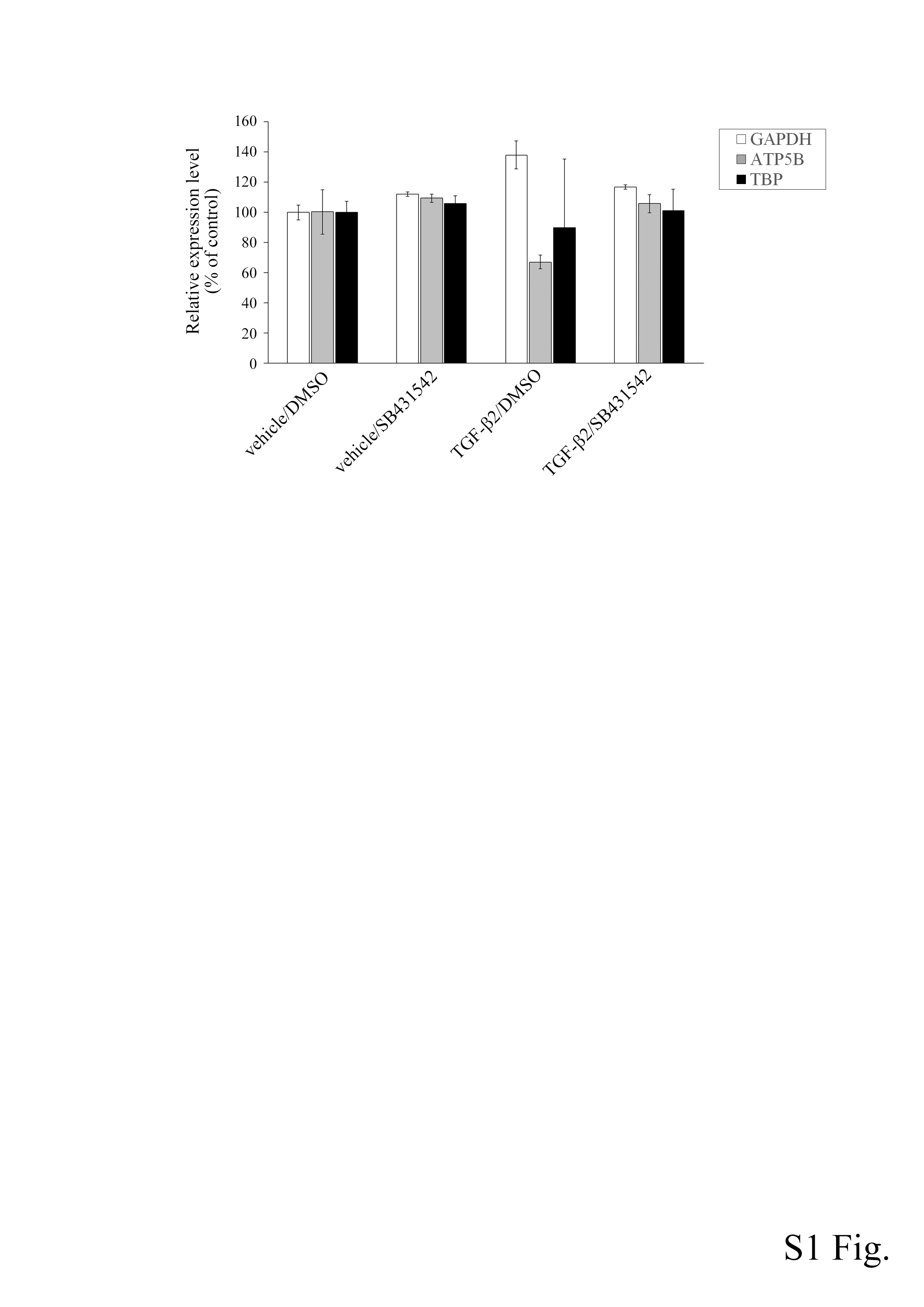

Supplement: S1 Fig — Expression level of three internal control genes in four RNA samples from A549 spheroids treated with 5 ng/mL TGF-β2 or the vehicle (0.1% BSA/4 mM HCL) and 10 μM SB431542 or DMSO were evaluated by qRT-PCR. TBP was the most stable gene out of 3 among 4 RNA samples. Data are mean ± SD of triplicate well. (TIF) [file pone.0162394.s001.tif]

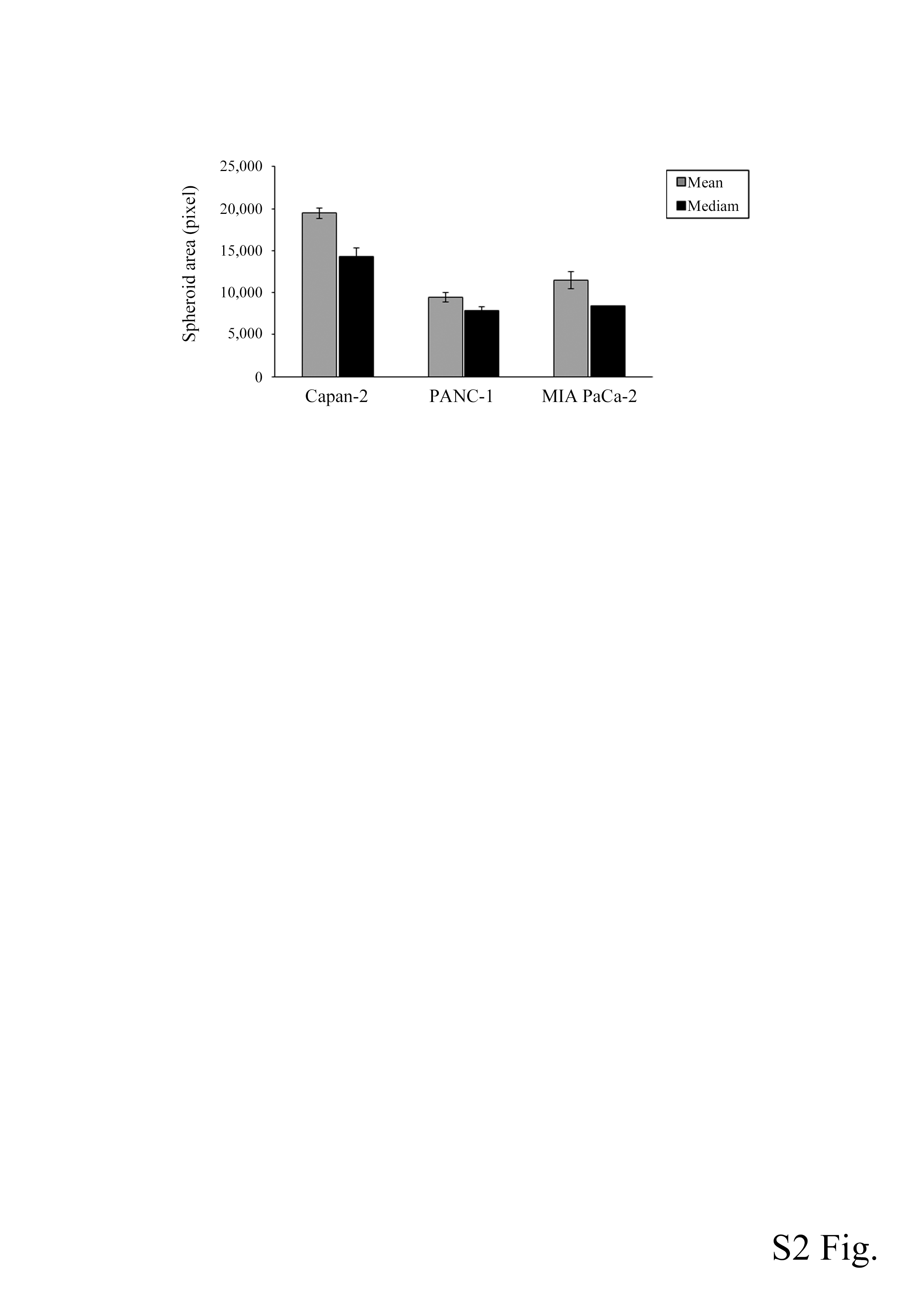

Supplement: S2 Fig — Three pancreas cancer cell lines were cultured on NCP and observed with Celigo for image analysis. Pixels of these spheroids in a whole well were measured as spheroid size with image J software, and then the mean and median were calculated. Data are mean ± SD of triplicate well. (TIF) [file pone.0162394.s002.tif]

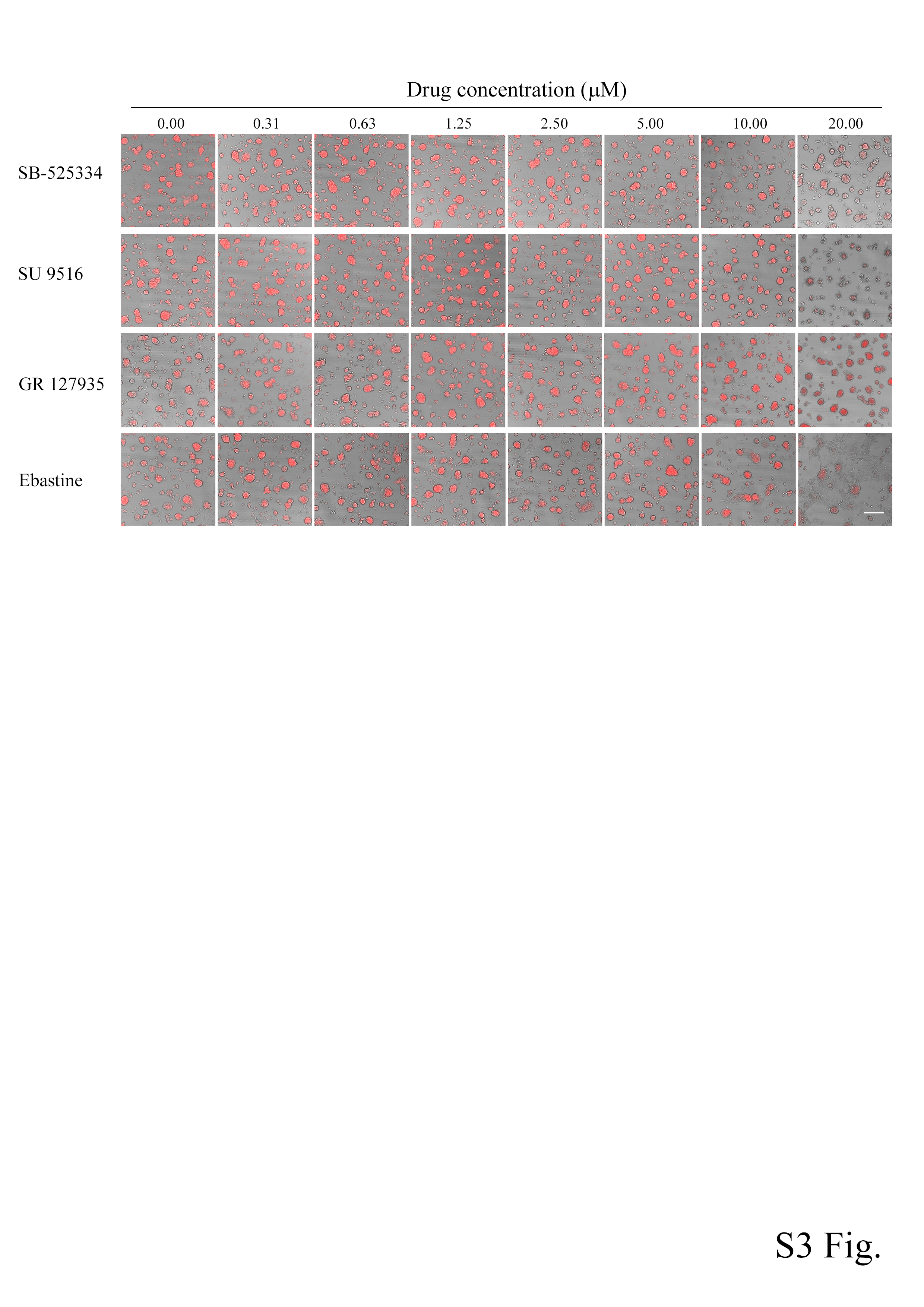

Supplement: S3 Fig — No EMT induction, only drug treated spheroids hypoxia images (red color). A549 spheroids cultured on NCP for 3 days were treated with each drugs at indicated concentrations for 4 days. In 20 μM SU 9516 treatment, spheroid sizes were small. (TIF) [file pone.0162394.s003.tif]
